# Temporal Patterns of Vital Signs and Their Association With 28-Day Survival in Patients With COVID-19-Associated Acute Respiratory Distress Syndrome (ARDS) Treated With High-Flow Nasal Oxygen: A Machine Learning Model

**DOI:** 10.7759/cureus.112509

**Published:** 2026-07-12

**Authors:** Rashid Nadeem, Zainab A Obaida, Sahish Kamat, Doaa El Gohary, Lamia Salama, Amr Awad, Atif Altafuddin Ahmed

**Affiliations:** 1 Intensive Care Medicine, Dubai Hospital, Dubai, ARE; 2 Data Science, University of Sharjah, Dubai, ARE

**Keywords:** acute respiratory distress syndrome (ards), covid-19, hfno, high-flow nasal oxygen, mechanical ventilation (mv), medical intensive care unit (micu), pulmonary disease, respiratory rate

## Abstract

Background: High-flow nasal oxygen (HFNO) is widely used in COVID-19-associated acute respiratory distress syndrome (ARDS) to delay or avoid invasive mechanical ventilation (MV). However, prolonged HFNO in the presence of worsening respiratory physiology may contribute to patient self-inflicted lung injury, and the optimal timing for escalation to MV remains uncertain. This study evaluated whether temporal patterns in vital signs and the duration of HFNO exposure were associated with 28-day mortality.

Methods: A retrospective observational study was conducted, including 98 adults with confirmed COVID-19 pneumonia admitted to the ICU on HFNO who subsequently required MV. Longitudinal vital signs were recorded three times daily for up to 28 days. Data were transformed into a patient-day panel to capture end-of-day clinical decision points. Feature engineering incorporated daily summaries, short-term trends, and persistence of abnormalities. Four modeling approaches-elastic-net logistic regression, discrete-time survival modeling, gradient boosting, and shallow decision trees-were trained using patient-grouped cross-validation. Model performance was assessed using precision-recall metrics and calibration.

Results: The cohort had a mean age of 61.8±13.9 years and a mean BMI of 28.5±6 kg/m²; overall mortality was approximately 80%, with 79 patients dying during hospitalization and 19 patients surviving to 28 days. Non-survivors exhibited persistently elevated respiratory rates, progressive declines in oxygen saturation, and greater variability in cardiovascular and respiratory vital signs. Divergence in respiratory rate between survivors and non-survivors became pronounced around day 12. BMI appeared to influence respiratory rate trajectories. Across all models, prolonged HFNO duration in the presence of worsening respiratory physiology was associated with increased mortality risk. Gradient boosting achieved the highest discrimination, while logistic regression and discrete-time survival models demonstrated superior calibration and interpretability.

Conclusion: Worsening respiratory trajectories-rather than isolated vital-sign abnormalities-were strongly associated with mortality among patients treated with HFNO prior to MV. The interaction between prolonged HFNO exposure and physiological deterioration suggests a time-sensitive escalation window during which intubation may improve outcomes. These findings are exploratory and require validation in larger, prospective cohorts to define actionable thresholds for the optimal timing of MV in COVID-19 ARDS.

## Introduction

High-flow nasal oxygen (HFNO) therapy has become a cornerstone of respiratory support for patients with acute hypoxemic respiratory failure, including those with COVID-19-associated acute respiratory distress syndrome (ARDS). Its ability to deliver heated, humidified oxygen at high flow rates has been shown to improve oxygenation, reduce the work of breathing, and, in many cases, delay or avoid the need for endotracheal intubation [1-3]. By reducing reliance on invasive mechanical ventilation (MV), HFNO may help mitigate complications such as ventilator-induced lung injury (VILI), ventilator-associated pneumonia, and hemodynamic instability, all of which are well-recognized risks of positive-pressure ventilation [4].

Despite these advantages, concerns have emerged regarding the potential for patient self-inflicted lung injury (pSILI) during prolonged HFNO use in patients with high respiratory drive. Experimental and clinical evidence suggests that vigorous spontaneous breathing, large tidal volumes, and excessive transpulmonary pressures may exacerbate lung injury and contribute to disease progression when respiratory support is insufficient to control respiratory effort [5-7]. This raises the possibility that HFNO, while beneficial early in the disease course, may become harmful if continued beyond a physiologically safe threshold.

The concept of an "optimal window" for HFNO use has therefore gained increasing attention. Several observational studies and expert reviews highlight the uncertainty surrounding the ideal timing for transitioning from HFNO to invasive MV, noting that both premature and delayed intubation may negatively affect outcomes [8,9]. Early intubation may expose patients to unnecessary risks of MV, whereas delayed intubation may allow ongoing pSILI, worsening hypoxemia, and multiorgan dysfunction. Identifying the point at which HFNO ceases to be protective and becomes injurious remains a critical unanswered question in the management of COVID-19-associated ARDS.

Traditional statistical approaches have struggled to define a clear threshold for safe HFNO duration, in part because the relationship between respiratory support, disease progression, and patient physiology is highly dynamic and nonlinear. Our previous work using conventional analytical methods did not identify a specific HFNO duration associated with improved survival outcomes. However, recent advances in artificial intelligence (AI) and machine-learning techniques offer new opportunities to analyze complex, longitudinal clinical data. Modern AI models can integrate continuous vital-sign trajectories, capturing nonlinear interactions and identifying subtle temporal patterns that may be overlooked by traditional methods [10-12].

In this study, we leveraged machine-learning approaches to examine the association between HFNO duration prior to invasive MV and survival outcomes in patients with COVID-19-associated ARDS. By incorporating 28-day longitudinal vital-sign data, we aimed to determine whether a maximal safe duration of HFNO can be identified and whether timely transition to invasive ventilation within this window is associated with improved clinical outcomes. This work seeks to address a critical gap in current clinical practice and may help guide more precise, individualized decisions regarding respiratory support escalation.

## Materials and methods

Study design and setting

This retrospective observational study included all patients with confirmed COVID-19 pneumonia who were admitted to the intensive care unit (ICU) at Dubai Hospital between January 1 and December 31, 2021, and who required invasive MV. Data were extracted from the Salama® electronic medical record system (Dubai Health, United Arab Emirates).

Study population

Eligible participants were adults aged 18 years or older with laboratory-confirmed COVID-19 pneumonia who underwent invasive MV during their ICU stay. Patients who did not require invasive MV, those younger than 18 years, and pregnant patients were excluded. Individuals with chronic lung diseases, including chronic obstructive pulmonary disease, asthma, and pulmonary fibrosis, as well as those with coronary artery disease or congestive heart failure, were included. For patients with multiple ICU admissions, only data from the index (first) ICU admission were analyzed.

Clinical management

The decision to initiate invasive MV was made by the treating clinicians based on bedside assessment. Indications for intubation included escalating respiratory distress, increased use of accessory muscles, persistent oxyhemoglobin desaturation, or hemodynamic instability. Oxygen therapy was titrated according to institutional protocol, with incremental increases in oxygen delivery when oxyhemoglobin saturation (SpO₂) fell below 90% on pulse oximetry. We used longitudinal physiological trajectories derived from repeated vital-sign measurements rather than single-time-point indices.

Variables and outcomes

The primary exposure variable was the duration of HFNO therapy prior to initiation of invasive MV, treated as a continuous variable. The primary outcome was 28-day survival from the time of ICU admission. Demographic variables included age, sex, and body mass index (BMI). Comorbidity data included diabetes mellitus, hypertension, coronary artery disease, chronic kidney disease, and outpatient dialysis dependence.

Data collection

Vital-sign data were collected at ICU admission and subsequently every eight-hour nursing shift for up to 28 days or until ICU discharge, whichever occurred first. Recorded parameters included temperature, heart rate, respiratory rate (RR), systolic and diastolic blood pressure, and oxyhemoglobin saturation. Additional clinical data collected at the time of ICU transfer included oxygen flow rate (L/min), use of invasive MV, vasopressor requirement, and inpatient dialysis status. All data were retrieved from the Salama® electronic medical record system (2017 version) and reviewed for completeness and accuracy prior to analysis. Medical records were de-identified before data extraction and analysis. Institutional Review Board (IRB) approval was obtained before study initiation.

Statistical analysis

The study cohort included 98 patients admitted to the ICU on HFNO, of whom approximately 80% died during hospitalization. All patients were followed longitudinally, with vital signs recorded three times daily for up to 28 days. To enable time-dependent modeling, the dataset was restructured into a patient-day panel format in which each row represented an end-of-day clinical decision point while the patient remained on HFNO and had not yet undergone intubation. This restructuring increased the number of analytic observations while preserving patient-level clustering to prevent information leakage.

Feature engineering focused on generating clinically interpretable temporal summaries. For each vital sign, end-of-day metrics, including minimum, maximum, mean, and range, were computed. Short-term physiological trends were captured using two- to three-day slopes and day-to-day changes [13]. Additional features quantified persistence and trajectory patterns, such as consecutive days of abnormal RR or oxygen saturation, as well as cumulative days on HFNO at each decision point [14]. Four modeling approaches were evaluated: regularized logistic regression using elastic-net penalties [15], a discrete-time survival model implemented as a logistic hazard function [16], gradient boosting [17], and a shallow, depth-restricted decision tree [18]. All models were trained and validated using patient-grouped cross-validation to ensure independence between the training and testing sets. Given the substantial class imbalance, model performance was assessed primarily using precision-recall metrics, supplemented by calibration curves and sensitivity analyses.

Across all modeling strategies, consistent physiological predictors of mortality emerged. Persistently elevated RR, particularly when increasing over consecutive days, was strongly associated with higher mortality risk among patients who remained on HFNO. Progressive declines in oxygen saturation over time, rather than isolated low values, were also reproducible predictors. Measures of physiological instability, reflected by widening daily ranges in respiratory and cardiovascular vital signs, further contributed to risk stratification. Notably, the combination of prolonged HFNO exposure and worsening respiratory physiology was associated with a marked increase in predicted mortality risk, suggesting the presence of a high-risk escalation window.

Among the evaluated models, gradient boosting demonstrated the highest discriminative performance, likely due to its ability to capture nonlinear interactions and complex temporal dependencies. However, regularized logistic regression and the discrete-time survival model exhibited comparable discrimination with superior calibration and interpretability and were therefore prioritized for inference. The shallow decision tree, while less discriminative, provided explicit and clinically interpretable decision rules that supported identification of deterioration thresholds based on multi-day patterns.

Given the modest sample size and pronounced outcome imbalance, these findings should be interpreted with caution. Although the models consistently identified deterioration trajectories associated with adverse outcomes when escalation was delayed, the results remain exploratory and hypothesis-generating. Larger, prospective, and more balanced datasets are needed to validate these findings and establish robust, generalizable thresholds for the optimal timing of invasive MV.

## Results

Baseline characteristics

**Figure 1:**
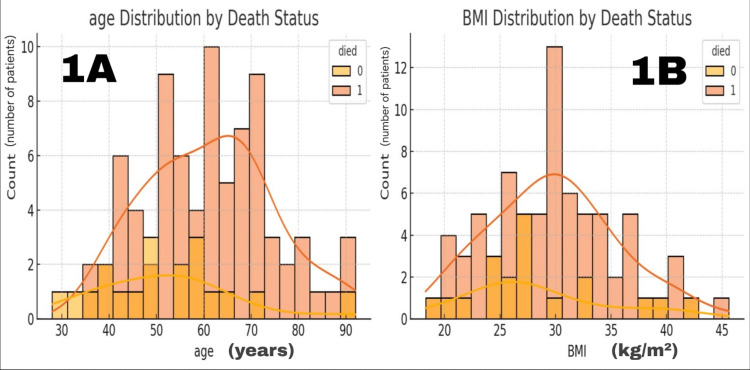
Age and BMI distributions stratified by 28-day survival status (A) Age distribution (years) among survivors and non-survivors. (B) BMI distribution (kg/m²) among survivors and non-survivors. BMI, body mass index

A total of 98 patients with confirmed COVID-19 pneumonia were included in the analysis. The mean age was 61.8±13.9 years, and the cohort consisted of 65 males and 33 females. The mean BMI was 28.5±6 kg/m². Overall mortality was high, with 79 patients (80%) dying during hospitalization and 19 patients surviving to 28 days. Figure 1A shows the age distribution according to survival status. Non-survivors tended to be older than survivors, with a greater concentration in the 55-75-year age range. Figure 1B shows the BMI distribution according to survival status. Non-survivors generally had higher BMI values, with the highest concentration around 30 kg/m².

Comorbidities were common in the cohort: diabetes mellitus was present in 63% of patients, hypertension in 48%, heart disease in 15%, and chronic renal failure in 23%.

Longitudinal vital‑sign patterns

**Figure 2:**
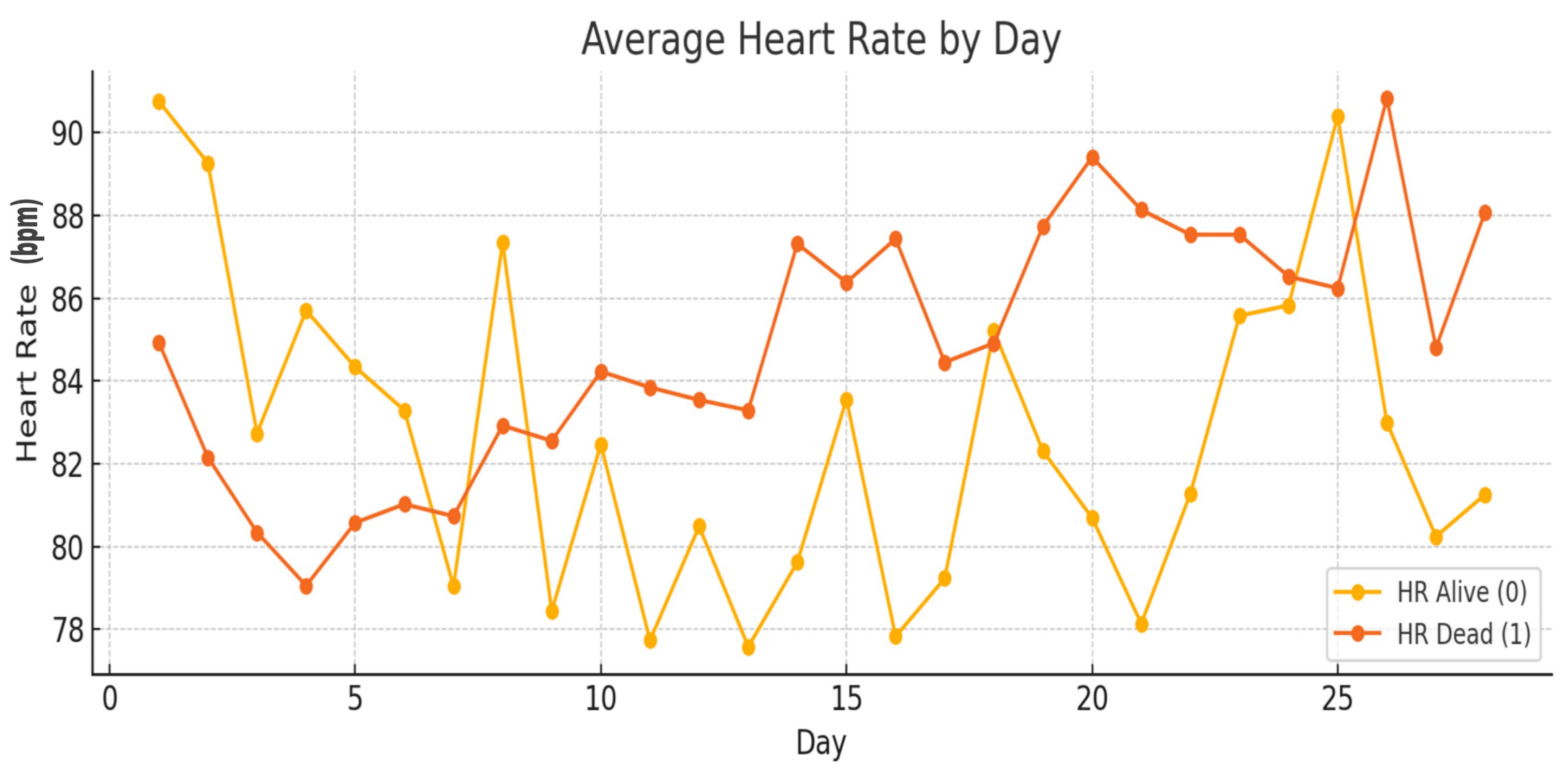
Mean daily heart rate over the 28-day study period, stratified by 28-day survival status HR, heart rate

Vital-sign trajectories over the 28-day observation period demonstrated clear physiological differences between survivors and non-survivors. Mean heart rate variability was consistently higher among non-survivors compared with survivors (Figure 2), reflecting greater cardiovascular instability.

**Figure 3:**
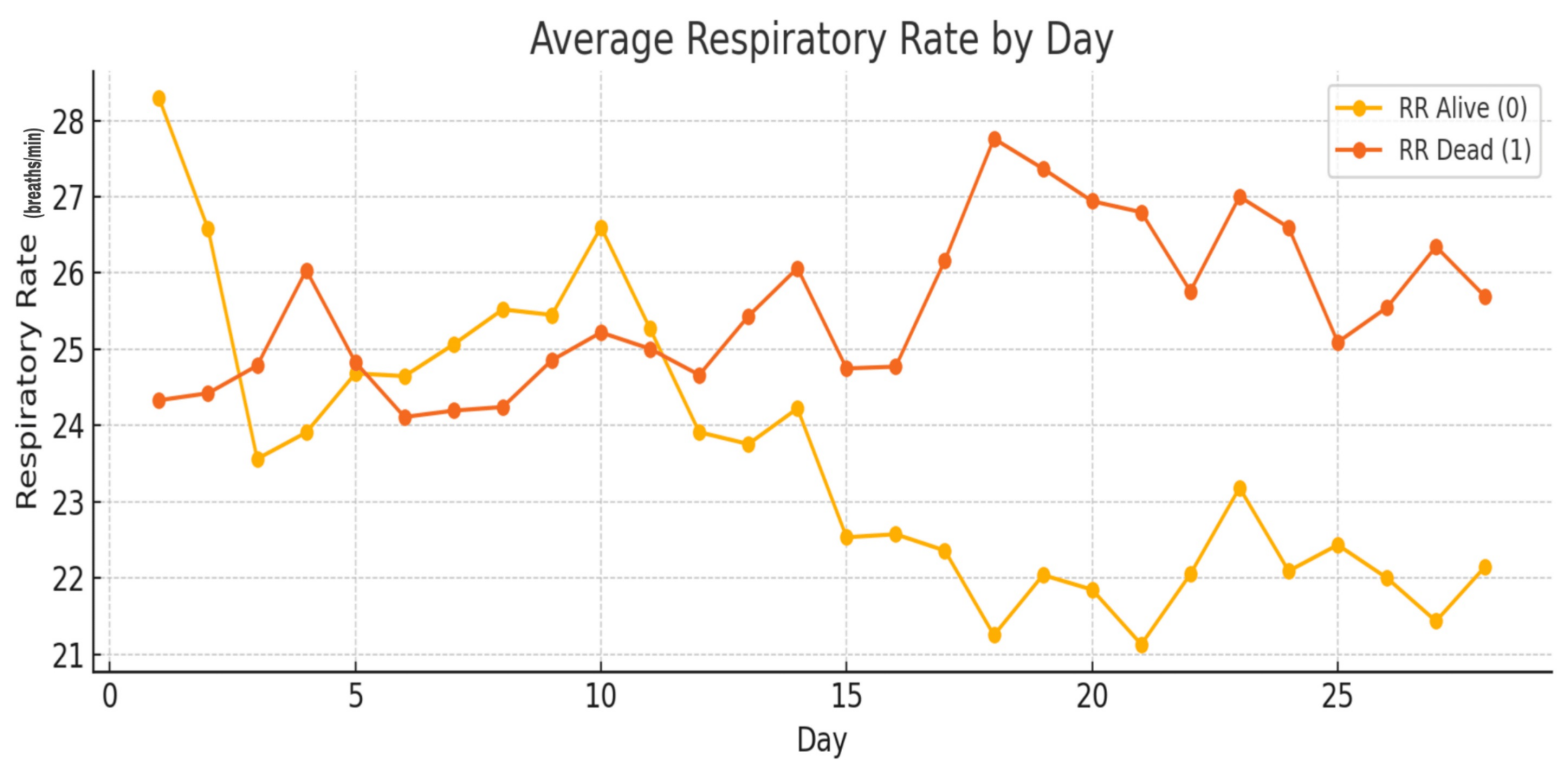
Mean daily RR over the 28-day study period, stratified by 28-day survival status. RR, respiratory rate

Similarly, the average RR was elevated in patients who died (Figure 3). Divergence in RR between survivors and non-survivors became more pronounced around day 12 of follow-up, suggesting progressive respiratory deterioration in those who ultimately died.

**Figure 4:**
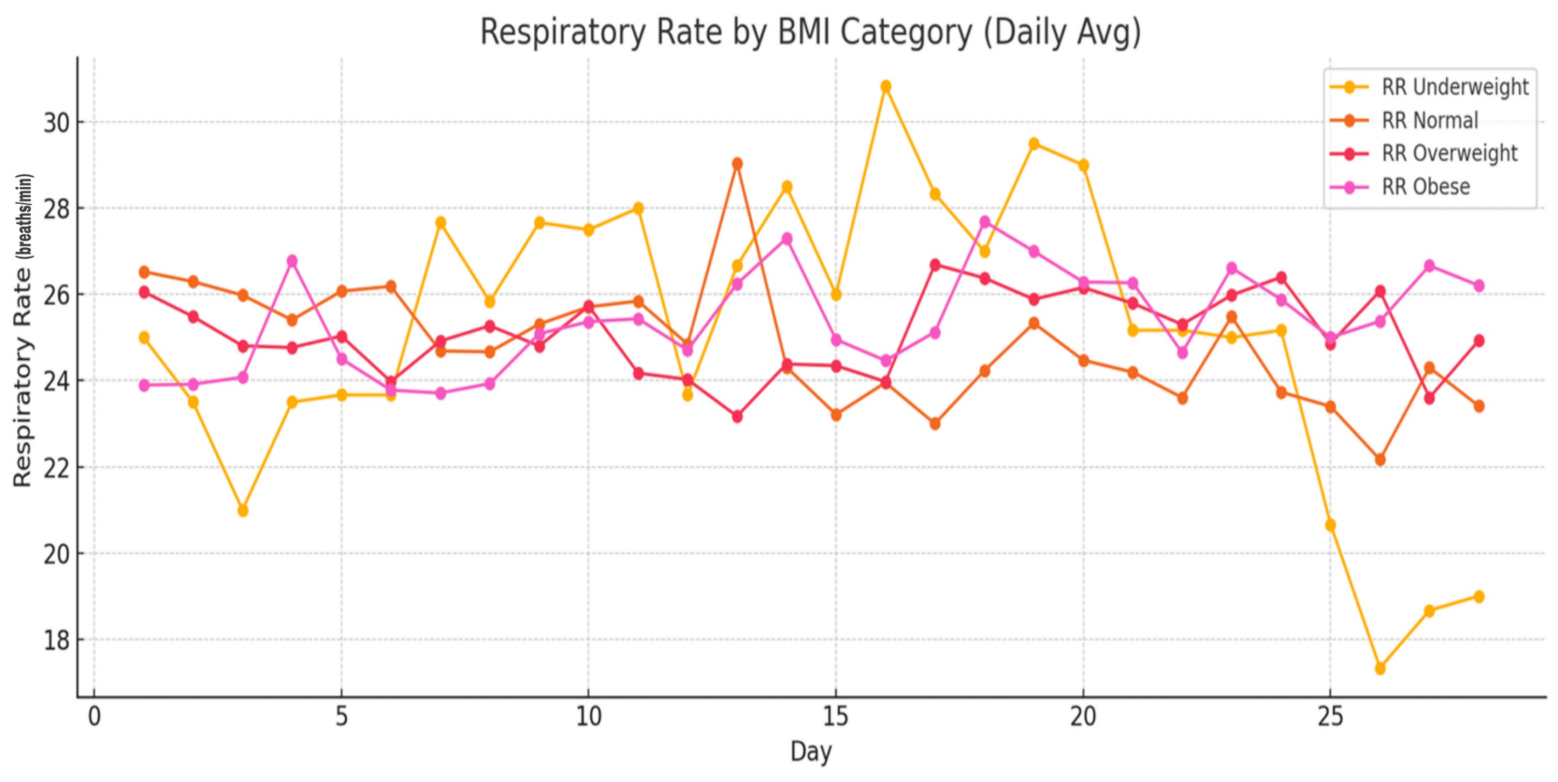
Mean daily RR according to BMI category RR, respiratory rate; BMI, body mass index

BMI also appeared to influence respiratory patterns: patients with higher BMI exhibited higher RRs throughout their ICU course compared with those with lower BMI (Figure 4).

## Discussion

In this retrospective cohort of critically ill patients with COVID-19-associated ARDS treated initially with HFNO, we observed a high overall mortality rate, consistent with reports from similarly severe populations. Several key physiological patterns emerged from the longitudinal data, suggesting that the trajectory of respiratory deterioration, rather than isolated measurements, may play a central role in determining outcomes. Our findings highlight the potential importance of identifying a time-sensitive escalation window during which transition from HFNO to invasive MV may be most beneficial.

A central observation was the strong association between persistently elevated RR and mortality. Non-survivors demonstrated higher RRs throughout their HFNO course, with divergence from survivors becoming more pronounced around day 12. This pattern aligns with the concept of pSILI, in which sustained high respiratory drive contributes to worsening lung injury and progressive hypoxemia [1-4]. The progressive decline in oxygen saturation observed among non-survivors further supports this mechanism, suggesting that prolonged spontaneous breathing under inadequate support may accelerate physiological deterioration.

Physiological instability also emerged as an important marker of risk. Non-survivors exhibited greater variability in heart rate and respiratory parameters, reflecting impaired compensatory capacity and heightened cardiorespiratory stress. These findings are consistent with prior studies demonstrating that widening variability in vital signs often precedes clinical decompensation in critically ill patients [13,14]. The influence of BMI on RR trajectories, whereby patients with higher BMI exhibited persistently higher RRs, may reflect the combined burden of obesity-related respiratory mechanics and COVID-19-associated lung injury [19-21].

Our machine-learning models consistently identified deterioration patterns that were strongly associated with mortality, regardless of the modeling approach. Gradient boosting achieved the highest discriminative performance, likely due to its ability to capture nonlinear interactions and complex temporal dependencies [15-18]. However, regularized logistic regression and discrete-time survival modeling demonstrated comparable performance with superior calibration and interpretability, making them more suitable for clinical inference. The shallow decision tree, while less discriminative, provided explicit and clinically intuitive rules that reinforced the importance of multi-day deterioration patterns.

Importantly, the interaction between HFNO duration and physiological decline suggests that prolonged HFNO use may be safe in patients with stable or improving respiratory parameters but potentially harmful in those with worsening trajectories. This finding supports the hypothesis that an optimal window for escalation to invasive MV may exist and that delaying intubation beyond this window, particularly in the presence of rising RR, declining oxygen saturation, or increasing physiological variability, may contribute to poorer outcomes [5-11]. These observations align with emerging literature emphasizing the risks of delayed intubation in COVID-19-associated ARDS and the potential for pSILI during prolonged HFNO therapy.

This study has several limitations. The sample size was modest, and the pronounced class imbalance limits the precision of the model estimates. As an observational study, residual confounding cannot be excluded, and clinical decisions regarding intubation were not standardized. Additionally, although the use of longitudinal vital-sign data strengthens the analysis, the absence of detailed ventilatory mechanics, imaging findings, and clinician-documented reasons for escalation limits the ability to fully characterize the decision-making process. Finally, the findings should be considered exploratory and hypothesis-generating rather than definitive. Despite these limitations, this study demonstrates the potential value of machine-learning approaches in identifying clinically meaningful deterioration patterns and informing escalation decisions in COVID-19-associated ARDS. The consistent association between worsening respiratory trajectories, prolonged HFNO exposure, and mortality underscores the need for prospective studies to validate these findings and define actionable, generalizable thresholds for timely intubation. Future work incorporating larger, more diverse cohorts and integrating additional physiological and imaging data may help refine predictive models and support real-time clinical decision-making. Information about outpatient treatment with ivermectin was not available, which may represent another limitation of the study.

## Conclusions

In this cohort of critically ill patients with COVID-19-associated ARDS treated initially with HFNO, worsening respiratory trajectories, rather than isolated vital-sign abnormalities, were strongly associated with mortality. Persistently elevated RR, progressive declines in oxygen saturation, and increasing physiological variability emerged as consistent predictors of adverse outcomes across multiple modeling approaches. The interaction between prolonged HFNO duration and deteriorating respiratory physiology suggests the presence of a time-sensitive escalation window during which transition to invasive MV may be most beneficial.

While these findings highlight clinically meaningful patterns, they remain exploratory given the modest sample size and substantial outcome imbalance. Larger, prospective studies incorporating richer physiological data are needed to validate these observations and define actionable, generalizable thresholds for the optimal timing of intubation. Nonetheless, this work demonstrates the potential value of machine-learning approaches in identifying early deterioration signals and supporting more precise, individualized escalation decisions in COVID-19-associated ARDS.
